# Characterization of the complete mitochondrial genome of the medical pipefish *Doryichthys boaja* Bleeker 1850

**DOI:** 10.1080/23802359.2018.1501303

**Published:** 2018-08-17

**Authors:** Yun Fang, Lingyan Zhu, Meng Chen, Yuqing Ge, Guangji Zhang, Rubin Cheng

**Affiliations:** a College of Pharmaceutical Science, Zhejiang Chinese Medical University, Hangzhou, P. R. China;; b The First Affiliated Hospital, Zhejiang Chinese Medical University, Hangzhou, P. R. China

**Keywords:** *Doryichthys boaja*, mitochondrial genome, evolutionary relationship, Syngnathidae family

## Abstract

In the present study, we reported the complete mitochondrial genome of medical pipefish *Doryichthys boaja* Bleeker 1850. The complete mitochondrial genome of *D. boaja* was 16,562 bp in length and had a base composition of A (31.10%), C (24.14%), G (14.36%), and T (30.40%). Similar to other Syngnathidae species, it contained a typically conserved structure, including 13 protein-coding genes, 2 rRNA genes, 1 control region (D-loop), and 22 tRNA genes. The 13 protein-coding genes encoded 3800 amino acids in total, most of which used the initiation codon ATG except CO1 gene started with GTG. For the stop codon, 6 genes applied TAA as the stop codon, while the other 7 genes used an incomplete stop codon T or TA. The lengths of 12S rRNA and 16S rRNA were 941 bp and 1671 bp, respectively. The control region of *D. boaja* ranged from 15,615 bp to 16,562 bp, which was 948 bp in length. The complete mitochondrial genome of *D. boaja* provided essential and important molecular data for phylogeography and evolutionary analysis of Syngnathidae Family.

*Doryichthys boaja* Bleeker 1850 (long-snouted pipefish) was a freshwater and estuarine pipefish species that occurred widely in Southeast Asia. It was one of the largest freshwater pipefishes, reaching up to 44 cm in length (Kuiter 2000). *Doryichthys boaja* was widely distributed in the streams, rivers, and brackish waters of south-east Asia. Since the overexploitation and habitat destruction, this species is now listed in the IUCN Red List of Threatened Species as Data Deficient. Similar to all other syngnathids, this species was ovoviviparous, and the males brood the embryos under their trunk prior to giving live birth (Wilson et al. 2001). Since there are morphological similarities between *D. boaja* and other *Syngnathidae* species, molecular studies based on mitochondrial DNA markers have proved to be valuable tools for species identification and phylogenetic relationship elucidation within the family. Compared with partial mtDNA sequences, complete mitochondrial genome with a large set of genes was generally considered as a useful tool for phylogenetic analysis and further development of species identification and restoration strategies (Cheng et al. 2013; Cheng, Fang, et al. 2017). Thus, it was useful to obtain the complete mitochondrial DNA of *D. boaja* and studying its phylogenetic position in Genus *Syngnathidae*, facilitating its genetic assay, greater management, and conservation.

Here, we sequenced and characterized the complete mitochondrial genome of *D. boaja*. The specimen of *D. boaja* was obtained from Chinese materia medica market in Anguo city of Hebei Province and identified based on its morphometric features, such as the six-cornered, with sharp trailing edges, and a spinous process (Liu et al. 2018). The sample of *D. boaja* (FY-26) was deposited in the collection centre of College of Pharmaceutical Science at Zhejiang Chinese Medical University. Samples used in this study were approved by Animal Ethics committee for experimentation, granted by Zhejiang Chinese Medical University. Total genomic DNA was extracted from the muscle tissue of tail using a Tiangen DNA extract kit (Tiangen Inc., Beijing, China), following the manufacturer’s instructions. The complete mtDNA of *D. boaja* was amplified and sequenced by 14 pairs of primers designed according to the published mitochondrial genome sequences in the genus *Hippocampus* (Cheng, Liao et al. 2017; Chen et al. 2017). The mitochondrial genome sequence of *D. boaja* with the annotated genes was deposited in GenBank under the accession number of MH259592. A total of 13 complete mitochondrial genomes in family Syngnathidae were collected and the phylogenetic relationships were inferred utilizing maximum-likelihood (ML) methods by MEGA 7.0 based on the concatenated supergene consisting of 13 mitochondrial protein-coding genes (Kumar et al. 2016).

The whole mitochondrial genome sequence of *D. boaja* had a circular genome of 16,562 bp, containing 13 protein-coding genes, 2 rRNA genes, 1 control region and 22 tRNA genes. The contents of A, C, G, and T were 31.10%, 24.14%, 14.36%, and 30.40%, respectively. AT and GC contents of mitochondrial genome were 61.50% and 38.50%, respectively. The protein coding and tRNA genes of the medical pipefish mitogenome were predicted using ARWEN (Laslett and Canback 2008). The proportion of coding sequences of *D. boaja* with a total length of 11,405 bp was 68.86%, which encoded 3800 amino acids. All protein-coding genes in *D. boaja* started with a typical ATG codon, except for COX1 that was initiated by a GTG start codon. For the stop codon, ND1, ATP8, ATP6, ND4L, ND5, and ND6 genes ended with complete TAA, while the other 7 genes terminated with a single base T or TA. Incomplete stop codon was also found in the mitochondrial genes of many other fish species (Yu and Kwak 2015; Zhu et al. 2018). The lengths of 12S ribosomal RNA and 16S ribosomal RNA were 941 bp and 1671 bp, respectively. The 22 tRNA genes varied from 67 to 74 bp in length. The tRNA-Ser gene contained a dihydrouridine arm replacement loop and the other 21 tRNA genes could be folded into the typical clover-leaf secondary structure. Similar with most vertebral mitochondrial genome, nucleotide overlaps and space gaps were very common in seahorses and pipefishes (Cheng et al. 2013; Ge et al. 2017). The control region locating between tRNA-Pro and tRNA-Phe gene was 948 bp in length, ranging from 15,615 to 16,562 bp.

Phylogenetic relationships among *D. boaja* and other 13 species with complete mitogenome sequences were constructed using *Solenostomus paradoxus* as outgroup. As shown in Figure 1, *D. boaja* clustered together with *Microphis brachyurus* with high statistical support, suggesting a relative close relationship between the genus *Doryichthys* and *Microphis*. The monophyletic group of *D. boaja* and *M. brachyurus* appears as sister to the clade of *Doryrhamphus japonicus*, indicating that these 3 species shared a more recent common ancestor than any other Syngnathidae species. Another medical pipefish *Syngnathoides biaculeatus* clustered with *Phycodurus eques*, while the clade of 5 *Hippocampus* species was placed as a sister relationship to *Corythoichthys flavofasciatus.* The data of *D. boaja* enriched the resource of Syngnathidae in systematic, population genetic, and evolutionary biological studies. When the mitogenome representing species from each order in Syngnathidae will be available, the relationship between the species in Syngnathidae will be more clear and complete.

**Figure 1.**
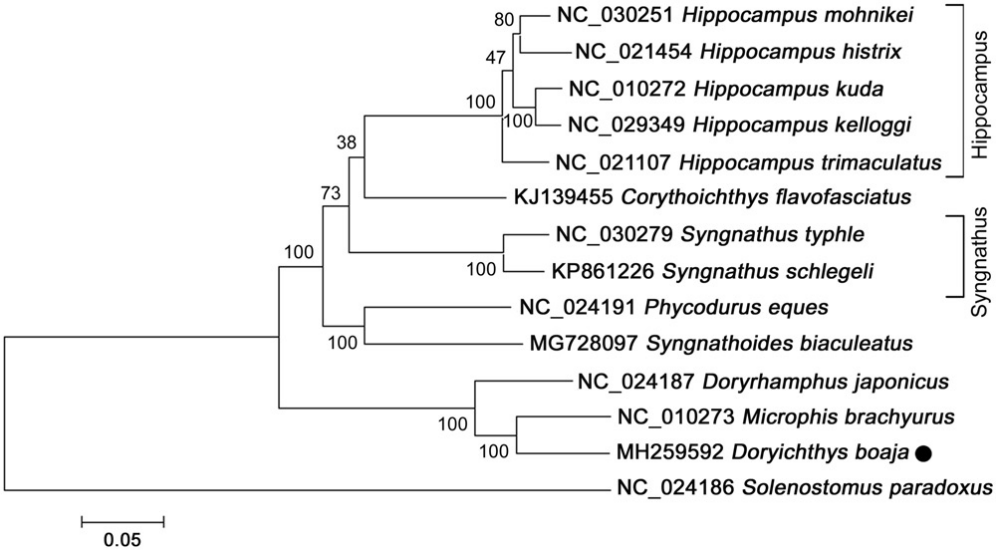
Neighbour-joining (NJ) tree of 14 species complete mitochondrial genome sequence. The phylogenetic relationships of *Doryichthys boaja* in Syngnathidae using *Sloenostomus paradoxus* as the outgroup. Number above each node indicates the ML bootstrap support values generated from 100 replicates.
